# The significance of matrix remodeling associated 7 (MXRA7) in pathogenesis or management of renal diseases deserves more investigations

**DOI:** 10.1080/0886022X.2024.2449575

**Published:** 2025-01-08

**Authors:** Yiqiang Wang

**Affiliations:** Wisdom Lake Academy of Pharmacy, Xi’an Jiaotong-Liverpool University, Suzhou, China

Dear Editor,

We read with great interest the article based on a research led by CKD Biomarkers Consortium, which tried to identify potential risk markers to be used for molecular diagnosis or drug development against chronic kidney diseases [1]. Starting from 4,638 plasma proteins in 3,235 participants of the Chronic Renal Insufficiency Cohort Study, Dubin et al. defined several batches of proteins with differential associations with disease status. Of specific interesting to us was that the matrix remodeling associated 7 (MXRA7) was among the eight proteins significantly associated with chronic kidney diseases (CKD) progression in Mendelian randomization analysis of GWAS datasets. Those authors claimed that those eight proteins as ‘potentially causal mediators of CKD progression that were significant after adjustment for multiple tests in one or more renal GWAS’. Regretfully, however, besides the single short sentence ‘MXRA7 is an extracellular matrix protein’ without any citation, not any other words were given for MXRA7, which appeared very important in the pathogenesis of CKD. We assumed that this negligence of MXRA7 was due to the rareness of the open publications that directly addressed the association of MXRA7 with kidney diseases or even with any topics on kidney. Actually, my lab might have been the sole team working on MXRA7 and published most of the papers designed on the biology of MXRA7 gene or its protein products. Collectively, the MXRA7 gene and its products were shown to play roles in ocular tissue development [2], bone marrow stromal cells or hematopoietic linages differentiation [3], chemical-induced liver injury [4], cutaneous wound healing [5], etc. The only obvious phenotype observed so far in MXRA7 knock-out mouse strain at either my site or by the International Mouse Phenotyping Consortium was femur shortening [3], implying that the molecular functions of MXRA7 are redundant in most physiological processes. Still, Dubin’s work added kidney to the organs list that might be associated with MXRA7 actions.

Inspired by Dubin’s findings, I looked for other clues buried in published papers or public datasets that contained information about MXRA7. In a study using a strategy similar to that of Dubin et al. Árnadóttir et al. found that MXRA7 ranked 31st (by adjusted P-value) among the 212 proteins manifesting significant correlations between serum levels and estimated glomerular filtration rates in CKD cohorts consisting of 5,764 individuals aged 66 - 96 [6]. By analyzing 255 microarray datasets of human glomerular and tubulointerstitial tissues for differentially expressed genes, Zhou et al. revealed that MXRA7 was among the genes up-regulated in diseased human glomerular and tubulointerstitial tissues with diabetic nephropathy [7]. By clustering analysis, it was further shown that the Gene Ontology terms containing MXRA7, i.e. ‘extracellular matrix (GO:0031012)’ or ‘collagen-containing extracellular matrix (GO:0062023)’, manifested the highest enrichment among all these regulated genes [7].

Besides CKD, acute kidney injury (AKI) seemed also to involve MXRA7 in certain to-be-defined manners. Prominent evidence was found by Paranjpe et al. in hospitalized SARS-CoV-2 patients, where plasma proteomics revealed 62 proteins associated with COVID-19-associated AKI, and MXRA7 was one of them. MXRA7 ranked 8th by coefficient value among the top 25 proteins that were significantly associated with estimated glomerular filtration rate after adjusting for age, sex, CKD history, etc. [8]. In another more unique context, namely kidney transplantation, van Leeuwen studied the proteins in the perfusate from the donor’s kidney during organ preparation, aiming at defining protein markers that might determine the deemed adequateness of the kidney for transplantation. It was found that higher contents of extracellular matrix proteins containing MXRA7 proteins in the perfusate obtained immediately before the ending of hypothermic machine perfusion predicted suboptimal outcomes (i.e. less transplantable) at one year after actual transplantation compared to lower contents of such proteins [9]. Just as those authors said, validating study with larger cohort was warranted to finalize a noninvasive biomarkers set to predict the graft outcome.

The third class of disastrous diseases involving MXRA7 is cancer. According to The Human Protein Atlas (HPA) [10], better survival outcomes were observed for 693 renal cancer patients with lower-than-cutoff MXRA7 expression compared with 184 patients with higher MXRA7 expression (*P* score = 0.0054), with 5-year survival rates of 71% vs 60% in these two groups respectively (as of July 8th, 2024). Possibly in line with the deleterious effect of MXRA7 expression changes (e.g. increase in most cases) with renal diseases, data in the HPA platform also manifested that MXRA7 was expressed in the kidney at low levels at either mRNA level or protein level, later of which were mainly restricted in glomeruli and absent in tubular areas.

In summary, by clearly revealing MXRA7’s role in CKD progress in large cohorts, Dubin et al. also proposed that MXRA7, together with several other novel proteins, might be exploited as drugable targets for CKD management. To us, we call for more attention from more scientists in more disease fields to work on this under-studied gene. Considering the fact that many genes including MXRA7 had been known by almost nothing, the report of Dubin et al. about the significance of MXRA7 in kidney physiology and related pathological processes were of value to the readers of Renal Failure as well.
